# Draft Genome Sequence of a Ketoprofen Degrader, Rhodococcus erythropolis IEGM 746

**DOI:** 10.1128/mra.01070-22

**Published:** 2022-11-16

**Authors:** Irina B. Ivshina, Elena A. Tyumina, Grigory A. Bazhutin, Maxim А. Polygalov, Anastasiia V. Krivoruchko

**Affiliations:** a Perm Federal Research Centre, Perm, Russia; University of Southern California

## Abstract

We report a draft genome sequence of Rhodococcus erythropolis IEGM 746 isolated from oil-polluted soil from an oil-extracting enterprise, Udmurt Republic, Russia. This strain was able to degrade ketoprofen, a commonly used nonsteroidal anti-inflammatory drug. Using the obtained sequence, putative genes encoding enzymes for ketoprofen degradation were revealed.

## ANNOUNCEMENT

Ketoprofen [KTP; C_16_H_14_O_3_; CAS 22071-15-4; 2-(3-benzoylphenyl)propanoic acid] is a commonly used nonsteroidal anti-inflammatory drug. Entering the environment, this biologically active compound becomes an emerging micropollutant with significant toxicological effects against biota (1, 2). Biological degradation plays the key role in removal of ketoprofen from the environment (3–5); however, this process is not fully elucidated. The Rhodococcus erythropolis strain IEGM 746 was isolated from oil-polluted soil near an oil-extracting enterprise, Udmurt Republic, Russia, and deposited in the Regional Specialized Collection of Alkanotrophic Microorganisms (acronym IEGM, number 768 WDCM, http://www.iegmcol.ru/strains/rhodoc/eryth/r_eryth746.html). This strain degraded up to 38% ketoprofen at its initial concentration of 100 mg/L in a mineral salt medium supplemented with 0.1% *n-*hexadecane as a cosubstrate in 14 days (6).

DNA was isolated from the R. erythropolis IEGM 746 cells preliminarily grown in LB broth at 28°C and 160 rpm for 28 h using the MagMAX DNA Multi-Sample Ultra 2.0 kit (Thermo Fisher Scientific) and a KingFisher Flex automatic station (Thermo Fisher Scientific) following the manufacturer’s protocol. A paired-end library (2 × 100 nucleotides [nt]) was produced using Illumina DNA Prep and sequenced on an Illumina NovaSeq 6000 instrument. A total number of 18,564,421 reads was obtained. Demultiplexing of the sequenced reads was performed with Illumina bcl2fastq (2.20). Adapters were trimmed with Skewer (version 0.2.2) (7). The quality of the FASTQ files was analyzed with FastQC (version 0.11.5-cegat) (8). The data were assembled with SPAdes v. 3.14.1 (9) at the scaffold level. Assembly statistics were computed using QUAST 5.2.0 (10). The assembly consisted of 78 contigs with a total sequence length of 6,772,728 bp, an *N*_50_ value of 516,745 bp, a GC content of 68.02%, and coverage of 275×. Average nucleotide identity (ANI) was calculated using the online tool https://www.ezbiocloud.net/tools/ani (11), and the highest OrthoANIu values of 95.38% and 98.74% were obtained for R. erythropolis (NCBI: txid1833) and Rhodococcus qingshengii (NCBI: txid334542) species consequently. Currently, *R. qingshengii* is a synonym of R. erythropolis (https://lpsn.dsmz.de/species/rhodococcus-qingshengii), and we consider the IEGM 746 strain as belonging to the R. erythropolis species. The annotation of coding sequences (CDS) was performed using PGAP 5.3 (annotation method “best-placed reference protein set,” GeneMarkS-2+) (12) and RAST 2.0 (annotation scheme RASTtk) (13). Default parameters were used for all software unless otherwise specified.

A total of 6,303 CDS, 6,249 CDS with protein, 3 rRNAs (5S, 16S, and 23S), and 52 tRNAs were found in the R. erythropolis IEGM 746 genome. Among CDS, those coding for monooxygenases (66 CDS), dioxygenases (28 CDS), proteins with decarboxylase activity (21 CDS), peroxidases (10 CDS), multicopper oxidases (5 CDS), vanillate *O*-demethylase oxidoreductases (EC 1.14.13.-) (3 CDS), and dehydrogenases (367 CDS) were revealed. Products of these genes can participate in ketoprofen degradation via oxidation, decarboxylation, or demethylation reactions.

### Data availability.

This Whole-Genome Shotgun project has been deposited in DDBJ/ENA/GenBank under the accession numbers JAJNDF010000001 to JAJNDF010000078. The version described in this paper is the first version, JAJNDF000000000.1. Raw sequence reads were deposited in the Sequence Read Archive with accession number SRR17030444 under BioSample accession number SAMN23420022 and BioProject accession number PRJNA783162.
